# Glucocorticoids impair oocyte developmental potential by triggering apoptosis of ovarian cells via activating the Fas system

**DOI:** 10.1038/srep24036

**Published:** 2016-04-04

**Authors:** Hong-Jie Yuan, Xiao Han, Nan He, Guo-Liang Wang, Shuai Gong, Juan Lin, Min Gao, Jing-He Tan

**Affiliations:** 1 College of Animal Science and Veterinary Medicine, Shandong Agricultural University, Tai-an City 271018, P. R. China

## Abstract

Previous studies indicate that stress damages oocytes with increased secretion of glucorticoids. However, although injection of female mice with cortisol decreased oocyte competence, exposure of mouse oocytes directly to physiological or stress-induced concentrations of glucorticoids did not affect oocyte maturation and embryo development. This study has explored the mechanisms by which glucocorticoids impair oocyte competence. Female mice were injected with cortisol and the effects of cortisol-injection on oocyte competence, ovarian cell apoptosis and Fas/FasL activation were observed. The results showed that cortisol-injection decreased (a) oocyte developmental potential, (b) the E2/P4 ratio in serum and ovaries, and (c) expression of insulin-like growth factor 1, brain-derived neurotrophic factor and glucocorticoid receptor in mural granulosa cells (MGCs), while increasing levels of (a) cortisol in serum and ovaries, (b) apoptosis in MGCs and cumulus cells (CCs), (c) FasL secretion in ovaries and during oocyte maturation *in vitro*, and (d) Fas in MGCs, CCs and oocytes. The detrimental effects of cortisol-injection on oocyte competence and apoptosis of MGCs and CCs were significantly relieved when the gld (generalized lymphoproliferative disorder) mice harboring FasL mutations were observed. Together, the results suggested that glucocorticoids impair oocyte competence by triggering apoptosis of ovarian cells via activating the Fas system.

Psychological stress can affect reproduction in women^1^,^2^. Restraint of small animals is an effective procedure to mimic human psychological stress^3^,^4^. Exposure of mice and rats to restraint stress during gestation impaired function of corpora lutea and decreased pregnancy rates and litter size^5^,^6^. However, evidences on the direct effect of psychological stress on the oocyte are limited. Although our recent studies showed that restraint stress of female mice impaired oocyte developmental potential^7^, the underlying mechanisms are largely unknown.

It is known that stress enhances the activity of the hypothalamus-pituitary-adrenal (HPA) axis and results in increased secretion of glucocorticoids from the adrenal cortex. For example, mice and rats exposed to stress showed significant elevation of serum corticosterone^8^ and cortisol^7^,^8^. However, although injection of female mice with cortisol decreased oocyte developmental potential^7^, exposure of mouse oocytes directly to physiological or stress-induced concentrations of cortisol^7^,^9^ or corticosterone^10^ during *in vitro* maturation did not affect nuclear maturation and embryo development. Thus, the mechanism by which glucocorticoids damage the oocyte has yet to be studied.

It is known that Fas signaling can induce apoptosis in various cells and tissues^11^,^12^. The presence of Fas/FasL system in ovaries has been reported in different species including mice^13^, rat^14^ and human^15^. Heat stress activated the Fas/FasL system in sertoli cells^16^. The Fas and caspase-3/8 activity increased significantly in cardiomyocytes undergoing apoptosis after restraint stress of rats^17^. Activation of the Fas/FasL system was also observed during postovulatory oocyte aging^18^. Treatment of mice with dexamethasone significantly increased FasL expression in testicular germ cells^19^. Glucocorticoids induced apoptosis in placenta cells^20^, testicular germ cells^21^ and Leydig cells^22^. Culture of osteocytes and monocytes with glucorticoids induced apoptosis with activation of the Fas/FasL system^23^,^24^. Furthermore, restraint stress diminished oocyte developmental potential by inducing apoptosis of ovarian cells^25^.

We thus hypothesized that glucocorticoids might impair oocyte developmental potential by triggering apoptosis of ovarian cells via activating the Fas system. The objective of this study was to test this hypothesis. Both wild-type mice and the gld (generalized lymphoproliferative disorder) mice that harbor FasL mutations were injected with cortisol, and the effects of cortisol injection on oocyte competence, ovarian cell apoptosis and Fas/FasL activation were observed. Cortisol was used instead of corticosterone because of the following reasons: (1) mouse serum cortisol and corticosterone are closely correlated in dynamics under different physiological or stressful conditions and both can be interchangeably used as indicators for rodent activation of stress^8^; (2) cortisol exhibits much higher glucocorticoid potency than corticosterone does^26^; (3) cortisol is cleared less rapidly than corticosterone is^27^; and (4) results obtained with cortisol will be more referable for studies on human beings because cortisol is the main glucorticoids in human.

## Results

### Injection of female mice with cortisol decreased oocyte developmental potential while increasing serum and ovarian cortisol levels

**Figure 1:**
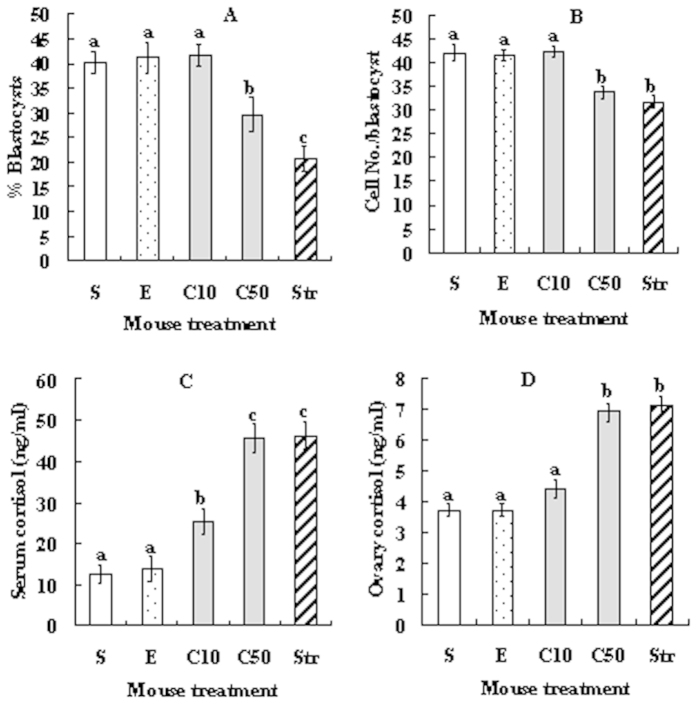
Effects of cortisol injection on blastocyst development of Sr^2+^-activated oocytes and on cortisol levels in serum and ovarian homogenates. (**A**) % Blastocysts; (**B**) Cell number per blastocyst; (**C**) Cortisol in serum; and (**D**) Cortisol in ovary. At 24 h after eCG injection, experimental mice were injected with cortisol at 10 (C10) or 50 (C50) mg/kg body weight, control mice were injected with either saline (S) or ethanol (**E**), and stressed control (Str) mice were restrained for 24 h. For oocyte maturation, each treatment was repeated 5 times with each replicate containing about 30–35 oocytes from 2 mice. For cortisol assays, each treatment was repeated 3 times with each replicate containing ovarian homogenates or serum from 3 mice. a–c: Values without a common letter above bars differ significantly (P < 0.05).

At 24 h after cortisol injection, oocytes at the germinal vesicle (GV) stage were recovered for *in vitro* maturation while blood and ovaries were collected for cortisol assay. Although rates for oocyte nuclear maturation (ranging from 96% to 98%) and activation (around 97%) did not differ between treatments, blastocyst rates and cell number per blastocyst decreased significantly when the cortisol dosage increased to 50 mg/kg bodyweight (Fig. 1). The blastocyst rate was significantly lower in oocytes from restraint stressed mice than that from mice injected with 50 mg/kg cortisol. Cortisol levels in both serum and ovaries were similar between mice injected with 50 mg/kg cortisol and the stressed mice but were significantly higher than that in control mice injected with saline or ethanol (Fig. 1). The results suggested that cortisol injection decreased oocyte developmental potential while increasing cortisol levels, and the effect was significant only when the injection dosage increased to 50 mg/kg. Although restraint stress and cortisol injection increased cortisol to the same level, the former produced significantly less blastocysts than the latter, suggesting that stress damages oocytes not only by raising glucorticoids but also by other means.

### Effects of cortisol injection on apoptosis of mural granulosa cells (MGCs) and cumulus cells (CCs)

**Figure 2:**
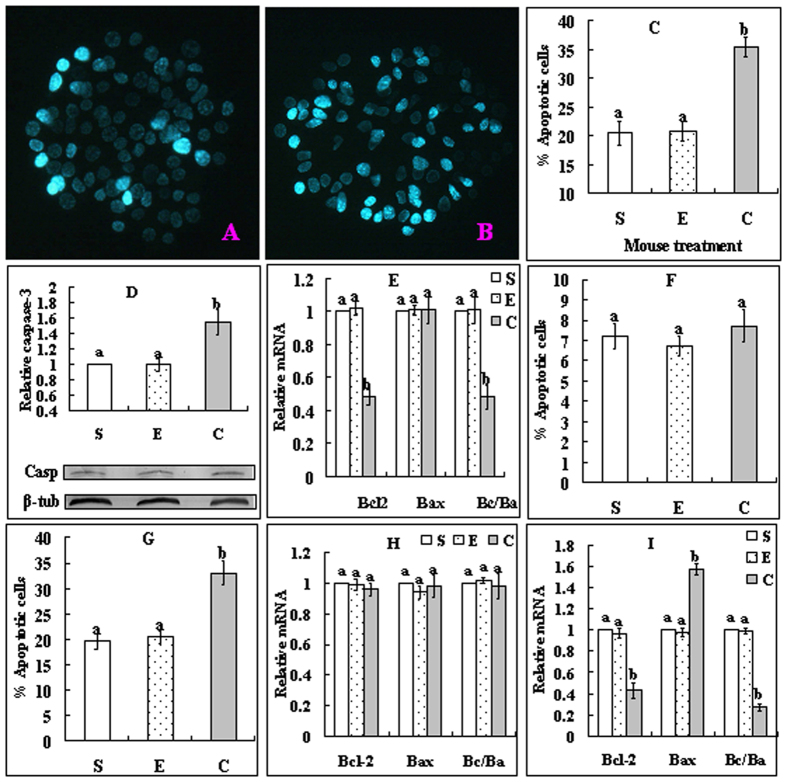
Effects of cortisol injection on apoptosis of MGCs (**A–E**) and CCs (**F–I**). Female mice were injected with saline (S), ethanol (**E**) or 50 mg/kg cortisol (**C**). A and B are MGCs smears stained with Hoechst 33342 and observed under a fluorescence microscope (Original magnification ×400), which show MGCs from mice injected with saline or cortisol, respectively. The heterochromatin is heavily stained with the Hoechst dye and gives bright fluorescence. Whereas the apoptotic cells show pyknotic nuclei full of heterochromatin, healthy cells show normal nuclei with sparse heterochromatin spots. Graph C shows percentages of apoptotic MGCs after different mouse treatments. Graph D shows levels of active caspase-3 while graph E shows Bcl2 and Bax mRNAs and Bcl2/Bax (Bc/Ba) ratio in MGCs after different mouse treatments. Graphs F and G show percentages of apoptotic CCs while graphs H and I show levels of Bcl2 and Bax mRNAs and Bcl2/Bax ratio in CCs, before and after oocyte culture without SGH, respectively. In all the experiments, each treatment was repeated 3 times. For the MGC experiments, each replicate included MGCs from 2 mice. For CCs Hoechst staining, each replicate contained 60 oocytes from 2 mice. For RT-PCR with CCs, each replicate contained CCs from 200 oocytes from 6–7 mice. a,b: Values without a common letter above their bars differ significantly (P < 0.05) within apoptotic markers.

At 24 h after cortisol injection, MGCs and oocytes were collected for assays of apoptotic markers. In MGCs, cortisol injection significantly increased the proportions of apoptotic cells (Fig. 2A–C) and the level of active caspase 3 (Fig. 2D), while decreasing Bcl-2 mRNA expression and the Bcl-2/Bax ratio (Fig. 2E). To observe apoptosis in CCs, while some of the oocytes were used for CCs recovery immediately after collection, others were cultured for 14 h in TCM-199 without serum, growth factors and hormones (SGH) before CCs harvest. Before culture, the percentage of apoptotic CCs did not differ between treatments (Fig. 2F), but after culture, it increased significantly in cortisol-injected mice (Fig. 2G). Before culture, neither Bcl-2 nor Bax mRNA expression differed among treatments (Fig. 2H), but after culture, while the level of Bcl-2 decreased, that of Bax increased significantly, leading to a significant decrease in the Bcl-2/Bax ratio in cortisol-injected mice (Fig. 2I). The results suggested that cortisol injection triggered apoptosis in MGCs, and it initiated an apoptosis program in the CCs during oocyte development in the ovary, which was executed later during oocyte maturation under unfavorable conditions.

### Effects of cortisol injection on levels of 17β-estradiol (E2), progesterone (P4), FasL and corticotropin-releasing hormone (CRH) in serum and ovarian homogenates

**Figure 3:**
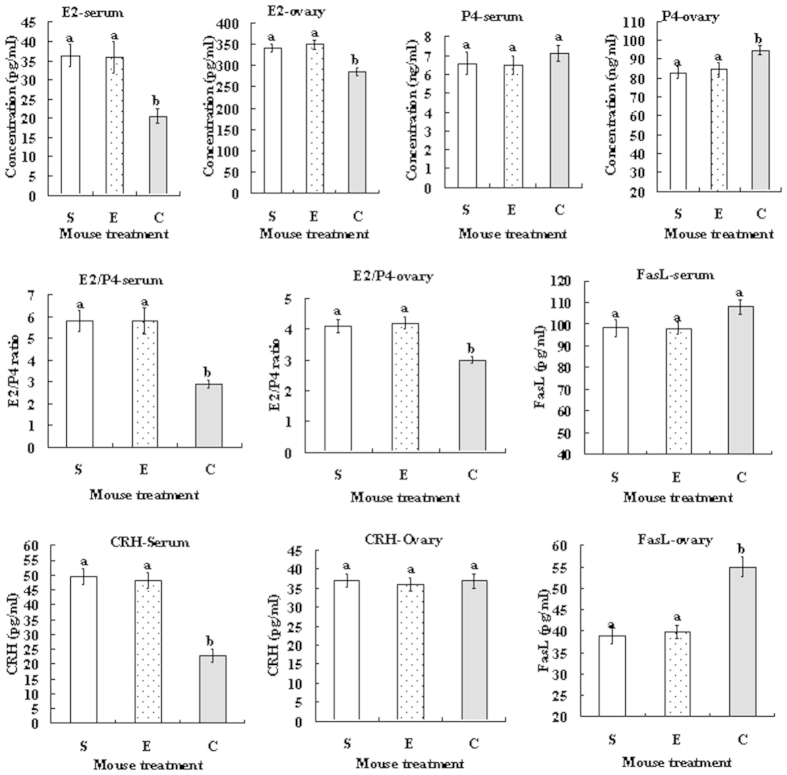
Effects of cortisol injection on contents of E2, P4, FasL and CRH and E2/P4 ratio in serum and ovarian homogenates. Female mice were injected with saline (S), ethanol (E) or 50 mg/kg cortisol (C) before assays. Each treatment was repeated 12 times with each replicate including serum or ovarian homogenates from a single (the same) mouse. a,b: Values without a common letter above their bars differ significantly (P < 0.05).

At 24 h after cortisol injection, blood and ovaries were collected for radioimmunoassay for E2 and P4 and for ELISA assay of FasL and CRH. In both serum and ovarian homogenates, whereas the level of E2 decreased, that of P4 increased in cortisol-injected mice compared to that in control mice (Fig. 3). Notably, the E2/P4 ratio in both serum and ovaries decreased significantly in cortisol-injected mice compared to that in control mice. Concentration of FasL in the ovary was significantly higher in cortisol-injected mice than in control mice, although that in serum did not differ significantly among treatments. Cortisol injection decreased the CRH level in serum significantly while having no effect on that in ovarian homogenates.

### Effects of cortisol injection on expression of insulin-like growth factor 1 (Igf1), brain-derived neurotrophic factor (Bdnf) and glucocorticoid receptor (Gr) mRNAs in MGCs

**Figure 4:**
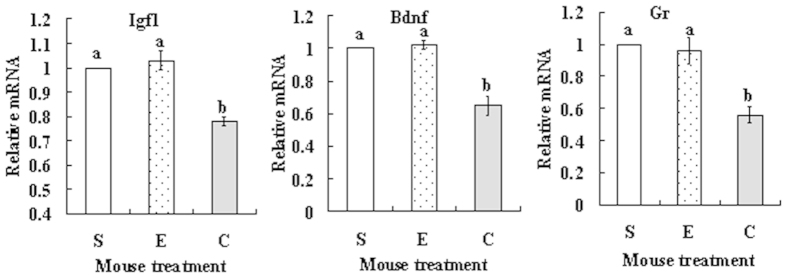
Effects of cortisol injection on expression of Igf-1, Bdnf and Gr mRNAs in MGCs. Female mice were injected with saline (S), ethanol (E) or 50 mg/kg cortisol (C) before real-time PCR assay for mRNA expression. Each treatment was repeated 3 times with each replicate including MGCs from 2 mice. a,b: Values without a common letter above their bars differ significantly (P < 0.05).

At 24 h after cortisol injection, MGCs were collected for real-time PCR assay. The relative levels of Igf1, Bdnf and Gr mRNAs in MGCs decreased significantly in cortisol-injected mice compared to that in control mice injected with saline or ethanol (Fig. 4).

### Effects of cortisol injection on Fas expression in oocytes, MGCs and CCs

**Figure 5:**
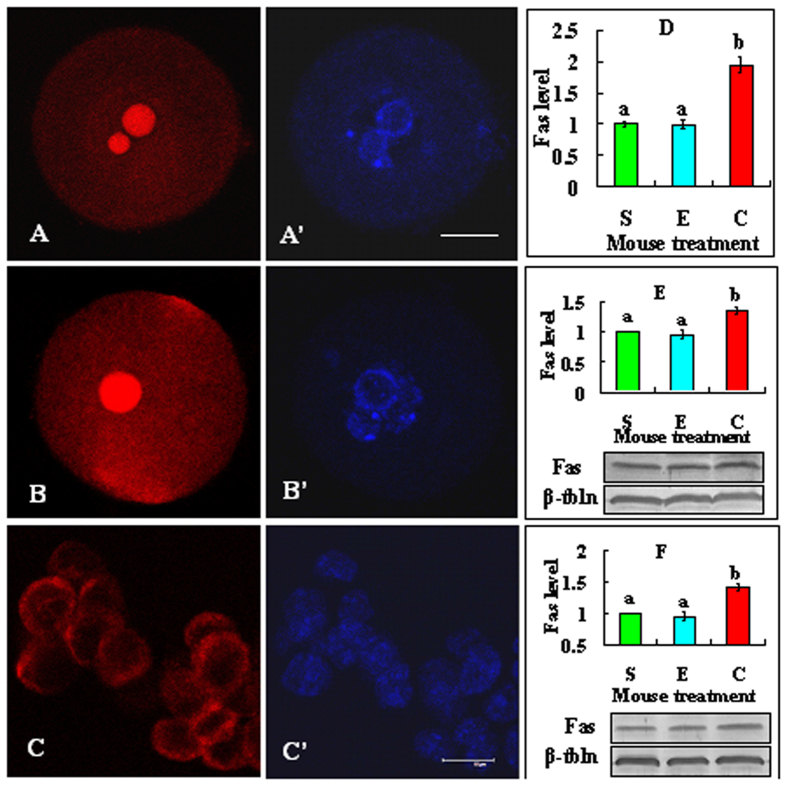
Effects of cortisol injection on Fas expression in oocytes, MGCs and CCs. Micrographs (**A–C**) are confocal images showing Fas localization in oocytes from saline- or cortisol-injected mice and in CCs, respectively. Images A and A’, B and B’, and C and C’ are the same samples observed under fluorescence after Fas and Hoechst staining, respectively. The bar is 20 μm and 10 μm in the oocyte picture and the CCs picture, respectively. Graph D shows Fas quantification in oocytes from mice injected with saline (S), ethanol (E) or 50 mg/kg cortisol (C). Each treatment was repeated 4 times with each replicate containing 20 oocytes. Graphs E and F show Fas quantification (Western blot analysis) in MGCs and CCs, respectively. Each treatment was repeated 3 times with each replicate including MGCs from 2 mice or CCs from 150 oocytes from 5 mice. a,b: Values without a common letter above their bars differ significantly (P < 0.05).

At 24 h after cortisol injection, oocytes at the GV stage and MGCs were recovered for analysis of Fas expression. After being freed of CCs, the denuded oocytes and some CCs were processed for immunofluorescence microscopy, whereas the MGCs and other CCs recovered were used for western blotting. Although Fas was localized mainly on the cell membrane in CCs, it was distributed not only on the plasma membrane but also throughout the cytoplasm and particularly concentrated in the nucleolus area in the oocyte (Fig. 5A–C). Quantification showed that cortisol injection significantly increased Fas expression in GV oocytes (Fig. 5D), MGCs (Fig. 5E) and CCs (Fig. 5F). Like what we observed in CCs in this study, Fas is usually localized on the plasma membrane in somatic cells^28^. Different from what we observed in mouse oocytes, however, no Fas expression was observed on the nucleus (GV) in bovine oocytes, although it was distributed at the cellular membrane and its adjacent cytoplasmic region^29^. Thus, the significance of Fas localization on the GV in mouse oocytes needs further investigations.

### Oocytes from cortisol-injected mice produced more FasL and were less competent than oocytes from ethanol-injected mice after maturation in TCM-199 without SGH

Oocytes at the GV stage recovered at 24 h after cortisol injection were cultured for maturation in TCM-199 without SGH. At 14 h of maturation culture, oocytes were freed of CCs to observe maturation rate, whereas the conditioned medium (CM) was collected for assay for FasL concentrations. Percentages of oocytes extruding first polar bodies were significantly (P < 0.05) lower in mice injected with cortisol (84.8 ± 3.8%, n = 150) than in control mice injected with saline (96.6 ± 1.0%, n = 150) or ethanol (96.9 ± 1.0%, n = 150). In contrast, concentrations of FasL in CM were significantly (P < 0.05) higher in mice injected with cortisol (172.4 ± 3.3 pg/ml) than in control mice injected with saline (156.4 ± 3.9 pg/ml) or ethanol (155.0 ± 6.2 pg/ml). The results suggested that cortisol-injection impaired oocyte competence by facilitating CCs production of FasL.

### Effects of cortisol injection on oocyte developmental potential and apoptosis of MGCs and CCs in gld mice

**Figure 6:**
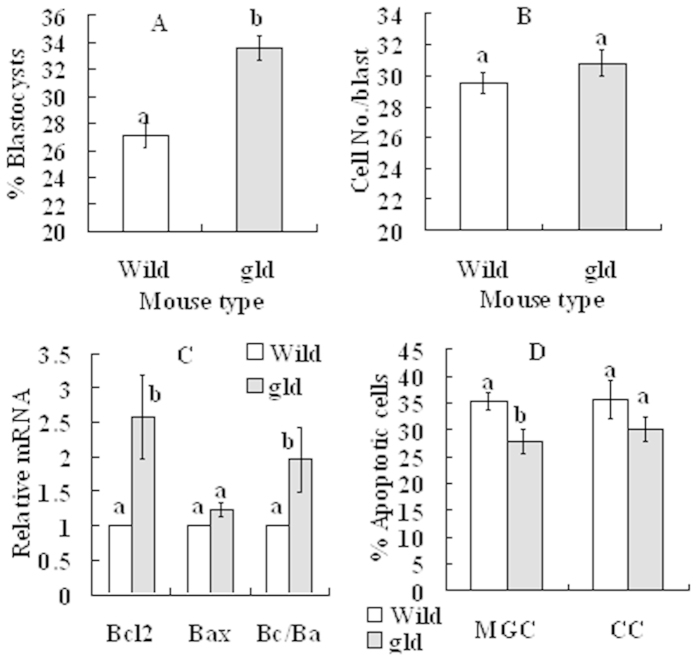
Effects of cortisol injection on blastocyst development and apoptosis in MGCs and CCs in wild-type mice and gld mice. (**A**) Rates of blastocysts; (**B**) Cell number per blastocyst; (**C**) Levels of Bcl2 and Bax mRNAs and Bcl2/Bax (Bc/Ba) ratio in MGCs; and D. Percentage of apoptotic MGCs and CCs after Hoechst staining. At 24 h after eCG injection, mice were injected with 50 mg/kg cortisol, and at 24 h after cortisol injection, mice were sacrificed to collect ovaries for further experiments. Percentages of blastocysts and cell number per blastocyst were observed after Sr^2+^-activation of oocytes. Apoptotic percentages of CCs were observed after oocytes were cultured for 14 h without SGH. For oocyte maturation for embryo development, each treatment was repeated 3 times with each replicate containing about 30–35 oocytes from 2 mice. In experiments with MGCs, each treatment was repeated 4 times with each replicate including MGCs from 3 mice. For Hoechst staining of CCs, each treatment was repeated 3 times with each replicate containing CCs from 30 oocytes from 3 mice. a,b: Values without a common letter above their bars differ significantly (P < 0.05).

Female gld mice and wild-type mice were injected with cortisol at 24 h after eCG injection, and at 24 h after cortisol injection, mice were sacrificed to collect ovaries for further analysis. Blastocyst rates were significantly higher in gld mice than in wild-type mice although the difference in cell number/blastocyst was not significant between mouse strains (Fig. 6A,B). Although levels of Bax mRNA in MGCs did not differ, levels of Bcl2 mRNA and the ratio of Bcl2/Bax mRNAs were significantly higher in gld mice than in wild-type mice (Fig. 6C). Apoptotic percentages of both MGCs and CCs were lower in gld mice than in wild-type mice (Fig. 6D), although the difference in CCs did not reach the level of statistical significance. The results further confirmed that glucocorticoids impaired oocyte developmental potential by causing apoptosis in MGCs and CCs through activating the Fas system.

## Discussion

The present results demonstrated that injecting female mice with cortisol down-regulated Gr mRNA expression in MGCs. Downregulation of GR expression by glucorticoids has been reported in other cell types. For example, whereas long-term adrenalectomy resulted in a large increase in whole-cell GR in rat brain, acute treatment with high dose corticosterone produced a large decrease in whole-cell GR^30^. Treatment of rats with dexamethasone resulted in a consistent decrease in the accumulation of Gr mRNA in all tissues studied including lung, spleen, brain, liver and kidney^31^. A progressive decrease in Gr mRNA was observed in the rat hippocampus with increasing doses of corticosterone^32^. Furthermore, dexamethasone caused a down-regulation of the levels of Gr mRNA and protein in hepatoma tissue culture cells^33^.

In this study, cortisol injection of female mice significantly increased FasL expression in ovaries and during oocyte maturation *in vitro*, and it promoted Fas expression in MGCs, CCs and oocytes. The detrimental effects of cortisol-injection on oocyte developmental potential and apoptotic parameters were significantly relieved when gld mice harboring FasL mutations were observed. The results suggested that glucorticoids impaired oocyte competence by activating the Fas signaling and triggering apoptosis in MGCs, CCs and oocytes. That stress induces apoptosis via activating the Fas system has been reported in different tissues and cells. Heat stress of mice activated the Fas/FasL system in sertoli cells^16^. Restraint stress of rats significantly increased the Fas and caspase-3/8 activity in apoptotic cardiomyocytes^17^. Treatment of mice with dexamethasone significantly increased FasL expression in testicular germ cells^19^. Culture with glucorticoids induced apoptosis with activation of the Fas/FasL system in osteocytes and monocytes^23^,^24^. Furthermore, GR binding to 2 negative glucorticoids regulatory elements (nGREs) in the FasL promoter reduces activation-induced FasL expression in T cells^34^. Together with our results that cortisol-injection reduced GR expression in MGCs, the data suggest that glucorticoids activate Fas signaling by down regulating GR expression.

The present results showed that cortisol injection decreased the E2/P4 ratio in serum and ovarian homogenates by reducing E2 while increasing P4 production. Studies have demonstrated that stress activates the HPA axis, and the HPA products affect ovarian function at the hypothalamus and the pituitary gland levels by decreasing the synthesis and release of LH and FSH. However, reports on the direct action of the HPA products on the ovary are few. *In vitro* studies showed that glucorticoids enhanced FSH-stimulated progesterone synthesis in cultured granulosa cells of rats and cattle^35^,^36^,^37^, and that glucorticoids suppressed P450 aromatase activity and decreased the number of LH receptors in rats^36^,^38^, cattle^37^ and pigs^39^. *In vivo* studies are even fewer. In cows, administration of ACTH increased plasma cortisol concentration while reducing 17β-estradiol concentrations in the follicular fluids^40^. In the protogynous Wrasse, cortisol administration induces sex change from ovary to testis and the plasma 17β-estradiol level was significantly lower in the cortisol treatment group than in the control group^41^. Furthermore, the level of estrogen is significantly reduced following chronic unpredictable stress or dexamethasone administration of mice supplemented with exogenous gonadotropin^42^.

The present results indicated that cortisol-injection of female mice reduced the expression of Igf1 and Bdnf mRNAs in MGCs. Short-term treatment of prepubertal mice with dexamethasone decreased growth and the IGF1 expression in the tibial growth plate^43^. Using cultures of rat osteoblasts, Delany and Canalis^44^ demonstrated that cortisol decreased Igf1 mRNA by approximately 50% through decreasing gene transcription. Furthermore, studies in the pig have shown that increased glucorticoid concentrations can disrupt ovarian IGF1 synthesis and IGF action both *in vitro*^45^ and *in vivo*^46^. In rats, both acute and chronic corticosterone administration significantly decreased Bdnf mRNA and protein in the hippocampus^47^,^48^. In mice, chronic unpredictable stress, which increased plasma corticosterone levels, significantly decreased BDNF expression in the antral follicles^49^. Furthermore, the GR-heterozygous mutant mice (GR+/−) with a 50% GR gene dose reduction show significant downregulation of BDNF protein content in their hippocampus^50^.

There are many reports that IGF1, BDNF and a high E2/P4 ratio are anti-apoptotic. For instance, estrogen, IGF1 and BDNF are all antiapoptotic in the ovary, particularly in granulosa cells^51^,^52^. In cattle, the healthy dominant follicles selected for ovulation enjoy a greater availability of IGF and a greater capacity to produce 17β-estradiol than do the subordinate follicles destined to undergo atresia^53^. In goats, healthy follicles showed significantly higher levels of 17β-estradiol and IGF1 than did the atretic follicles^51^. The level of progesterone, on the other hand, was higher in atretic follicles than in healthy follicles. The apoptotic percentage of goat granulosa cells cultured *in vitro* declined significantly in the presence of IGF1^54^. Furthermore, IGF1 exerts a stimulatory effect on aromatase activity and steroidogenesis in cultured rat granulosa cells^55^,^56^. In recent years, increasing evidence shows that ovarian BDNF plays an important role in oocyte development and maturation^57^,^58^,^59^. Notably, one study has shown that chronic unpredictable stress decreases the expression of BDNF in mouse ovaries^49^.

**Figure 7:**
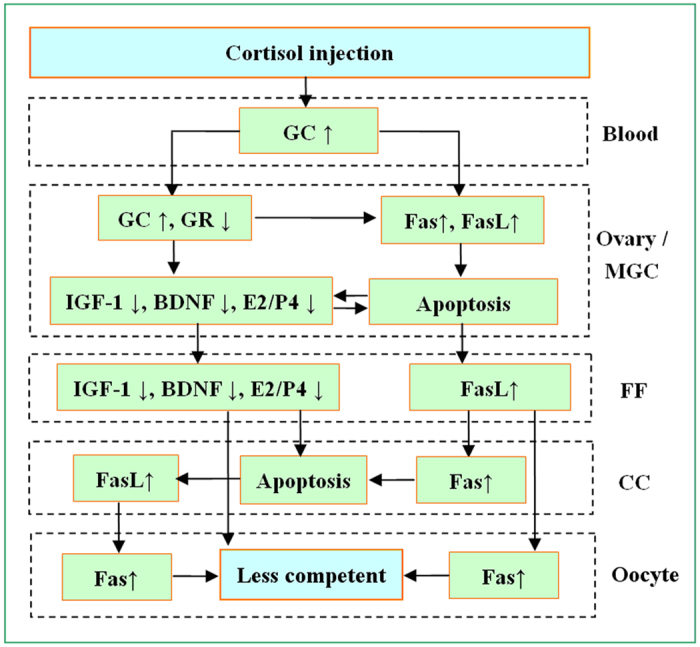
The possible pathways by which cortisol injection of female mice diminishes oocyte developmental competence. Cortisol injection increases the level of glucorticoids (GC) in blood and ovary. Within the ovary, the GC elevation activates the Fas/FasL system and decreases levels of growth factors and the E2/P4 ratio by down-regulating the expression of GR. The activation of Fas/FasL and the decrease in growth factors and E2/P4 ratio work together to induce apoptosis of ovarian cells, leading to a decline in growth factor levels and E2/P4 ratio but an increase in FasL in the follicular fluid (FF). The increase in FasL and the decrease in growth factor levels and the E2/P4 ratio in FF cause apoptosis in both CCs and oocytes, impairing oocyte developmental potential. Furthermore, the apoptotic CCs also produce FasL that affects oocytes by acting on their Fas receptors.

In summary, we have studied the mechanisms by which glucorticoids impair oocyte competence. Both wild-type mice and the gld mice that harbor FasL mutations were injected with cortisol and the effects of cortisol injection on oocyte competence, ovarian cell apoptosis and Fas/FasL activation were observed. The results indicated that cortisol injection increased the level of glucorticoids in blood and ovary (Fig. 7). Within the ovary, the elevation in glucorticoids activated the Fas/FasL system and decreased the levels of growth factors and the E2/P4 ratio by down-regulating the expression of GR. The activation of Fas/FasL and the decrease in growth factors and E2/P4 ratio worked together to induce apoptosis of ovarian cells, leading to a decline in growth factor levels and E2/P4 ratio, and an increase in FasL, in the follicular fluid. The increase in FasL and the decrease in growth factor levels and the E2/P4 ratio in follicular fluid cause apoptosis in both CCs and oocytes, impairing oocyte developmental potential. Furthermore, the apoptotic CCs also produce FasL that affects oocytes by acting on oocyte Fas receptors. The data have the first time up-to-date established the pathways by which glucorticoids diminishes oocyte developmental potential and are important for our understanding the role of glucocorticoids in a broad spectrum of stress-related diseases.

## Methods

The experimental procedures used for animal care and handling were approved by the Animal Care and Use Committee of the Shandong Agricultural University P. R. China (Permit number: SDAUA-2001-001). The methods were carried out in accordance with the approved guidelines. Unless otherwise specified, all chemicals and reagents used were purchased from Sigma Chemical Co. (St. Louis, MO, USA).

### Mice and treatment

Mice of the Kunming breed, which were used in most of the experiments, were bred in this laboratory. The gld mice harboring a germline mutation F273L in FasL with a C57BL/6J genomic background and the wild-type C57BL/6J mice were obtained from the Key Laboratory of Stem Cell Biology, Shanghai Institute for Biological Sciences, China. Mice were raised in rooms with a constant temperature between 22 and 25 °C and a photoperiod of 14 h light and 10 h dark with lights off at 20:00. Female mice, 8–10 weeks after birth, were injected with equine chorionic gonadotropin (eCG, 10 IU i.p.), and at 24 h after eCG injection, mice were injected with cortisol or subjected to restraint stress for 24 h.

Cortisol injection: Cortisol dissolved in 50% alcohol in saline was administrated intraperitoneally. Cortisol powder was first dissolved in absolute alcohol, and the stock solution of cortisol was then diluted with the same volume of saline before injection. To avoid oocyte damage by alcohol, the amount of solution injected was strictly controlled at less than 0.2 ml/animal. To this end, the amounts of cortisol powder to be dissolved in absolute alcohol were carefully calculated for different dosages. For example, to inject mice weighing 30 g with a dose of 50 mg/kg, a stock solution was prepared by dissolving 15 mg of cortisol powder in 1 ml of absolute alcohol. The 1-ml stock solution was then diluted with 1 ml of saline, with the resulting solution therefore containing 7.5 mg/ml (i.e., 1.5 mg/0.2 ml) of cortisol.

Restraint procedure: For restraint treatment, an individual mouse was put in a microcage constructed by the authors^11^, which was placed in an ordinary home cage. The microcage offered the same photoperiod and controlled temperature as in the large home cage for the unstressed animals. While in the microcage, mice could move back and forth to some extent and could take food and water freely, but they could not turn around.

### Recovery of ovaries, oocytes and mural granulosa cells (MGCs)

At 24 h after cortisol injection or restraint stress (48 h after eCG injection), mice were killed by decollation to collect ovaries for the recovery of oocytes and MGCs. The large follicles on the ovary were ruptured in M2 medium to release oocytes. Only oocytes with more than three layers of unexpanded cumulus cells (CCs), containing oocytes larger than 70 μm in diameter, and with a homogenous cytoplasm were used for experiments. The MGCs sheets released into M2 medium at puncture of follicles were collected for further use. CCs were freed from oocytes mechanically by pipetting oocytes in M2 medium. Both MGCs and CCs were pelleted by centrifugation at 500 × g for 5 min at room temperature. The pellets were then resuspended in Trizol (Invitrogen, Australia Pty. Ltd.) or RIPA lysis buffer for use in quantitative real-time PCR and western blot analysis, respectively.

### Oocyte maturation *in vitro*

The recovered oocytes were washed three times in M2 medium and once in maturation medium. The oocytes were then cultured in groups (30–35 oocytes) in 100 μl drops of maturation medium at 37.5 °C in a humidified atmosphere of 5% CO_2_ in air. The maturation medium was TCM-199 (Gibco) supplemented with 10% (v/v) fetal calf serum (Gibco), 1 μg/ml of 17β-estradiol, 24.2 mg/L of sodium pyruvate, 0.05 IU/ml of FSH, 0.05 IU/ml of LH, and 10 ng/ml of epidermal growth factor (EGF). In the maturation medium used to test CCs apoptosis, serum, growth factor and hormone (SGH) were omitted, and 24.2 mg/L of sodium pyruvate and 0.3 mg/ml of polyvinyl alcohol was supplemented.

### Oocyte activation and embryo culture

At 24 h of maturation culture, oocytes were stripped of their CCs by pipetting in M2 containing 0.1% hyaluronidase. After being washed twice in M2 and once in the activating medium (Ca^2+^-free CZB medium supplemented with 10 mM SrCl_2_ and 5 μg/ml cytochalasin B), the oocytes were incubated in the activating medium for 6 h at 37.5 °C in a humidified atmosphere with 5% CO_2_ in air. At the end of activation treatment, the oocytes were examined with a microscope for the evidence of activation. Oocytes were considered activated when each contained one or two well-developed pronuclei.

Activated oocytes were cultured for 4 days in regular CZB (about 30 oocytes per 100 μl drop) at 37.5 °C under humidified atmosphere with 5% CO_2_ in air. Glucose (5.5mM) was added to CZB when embryos were cultured beyond 3- or 4-cell stages. At the end of culture, embryo development was examined, and some of the blastocysts were stained with Hoechst 33342 for cell number counting.

### Blood serum preparation and ovarian homogenization

Mice were killed by decollation, and trunk blood was collected into ice-cooled centrifugal tubes and centrifuged (1700 × g, 10 min, 4 °C) to separate serum. The serum collected was stored at −80 °C until hormone assay. For ovarian homogenization, the ovaries were snap-frozen in liquid nitrogen immediately after removal from the mice. The frozen ovaries were weighed and transferred to an electrical homogenizer (ULTRA TURRAX IKA T18 basic) with the proper amount of homogenization solutions. Homogenization was performed while cooling on ice. Following homogenization, the homogenates were centrifuged (15000 × g, 10 min, 4 °C), and the supernatant was collected for immediate use or stored at −80 °C until use.

### Hormone assays

Ovaries were homogenized in PBS (800 μl per 100 mg of ovarian tissue). Radioimmunoassay was conducted by the Central Hospital of Tai-An City using commercial kits from Jiuding Biomedical Techniques Co. Ltd. The minimum levels of detection for assays of 17β-estradiol, progesterone, and cortisol were 1 pg/ml, 0.1 ng/ml, and 0.15 ng/ml, respectively. The intra- and inter-assay coefficients of variation were, respectively, 7.7% and 8.9% for 17β-estradiol, 7.2% and 8.9% for progesterone, and less than 10% for cortisol.

### Assessment of cell apoptosis by Hoechst 33342 staining

Hoechst staining was used to assess MGCs and CCs apoptosis because previous studies have shown that TUNEL and Hoechst staining are comparable methods for detecting apoptosis^60^. The MGCs obtained as described above and CCs released from 60–80 oocytes were dispersed by repeatedly pipetting using a thin pipette. The dispersed cells were centrifuged (200 × g) for 5 min at room temperature. The pellets were resuspended in 50 μl of M2 medium containing 10 μg/ml of Hoechst 33342 and stained in the dark for 5 min. The stained cells were resuspended in M2 and centrifuged at 200 × g twice (5 min each). Finally, a 5-μl drop of suspension was smeared on a slide and examined under a Leica DMLB fluorescence microscope (400×). Six to eight fields were randomly observed on each smear, and the percentages of apoptotic cells were calculated double blindly by 2 investigators.

### Western blotting

MGCs from 2 mice and CCs from about 150 oocytes from 5 mice were placed in a 1.5 ml microfuge tube containing 20 μl sample buffer (20 mM Hepes, 100 mM KCl, 5 mM MgCl_2_, 2 mM DTT, 0.3 mM phenylmethyl sulfonyl fluoride, 3 μg/mL leupetin, pH 7.5) and frozen at −80 °C. For running the gel, 5μl of 5 × SDS-PAGE loading buffer was added to each tube, and the tubes were heated to 100 °C for 5 min. Total proteins were separated on a 15% polyacrylamide gel by SDS-PAGE and transferred electrophoretically onto PVDF membranes. After being washed in TBST (150 mM NaCl, 2 mM KCl, 25 mM Tris, 0.05% Tween 20, pH 7.4) and blocked with TBST containing 3% BSA for 1 h at 37 °C, the membranes were incubated at 4 °C overnight with rabbit anti-active Caspase 3 (Casp3) polyclonal antibodies (1:1000, ab13847, Abcam Co. Ltd.) or rabbit anti-Fas (1:1000, ab82419, Abcam Co. Ltd.) and mouse anti-β-tubulin monoclonal antibodies (1:1000, 05-661, Merck Millipore). Then, the membranes were washed in TBST and incubated for 1 h at 37 °C with alkaline phosphatase-conjugated goat anti-rabbit IgG (1:1000, cw0111, Kangweishiji Biotechnology Co. Ltd., Beijing, China) and goat anti-mouse IgG (1:1000, cw0110, Kangweishiji Biotechnology Co. Ltd., Beijing, China). Finally, signals were detected by a BCIP/NBT alkaline phosphatase color development kit (Beyotime Institute of Biotechnology, Haimen City, China). The relative quantities of proteins were determined with Image J software by analyzing the sum density of each protein band image. The relative quantity values of active-Casp3 and Fas in control mice injected with saline were set as one and the other values were expressed relative to this quantity.

### Quantitative real-time PCR

Ovarian homogenization was performed using Trizol reagent (0.5 ml per ovary). MGCs from two mice and CCs from about 200 oocytes were treated with Trizol reagent for RNA isolation. The RNA isolated was resuspended in diethylpyrocarbonate-treated MilliQ water (DEPC-dH_2_O). The purified RNA was dissolved in DEPC -dH_2_O and spectroscopically quantified at 260 nm. Purity and integrity of the RNA was assessed by determination of the A_260_/A_280_ ratio (1.8–2.0) and electrophoresis in 1% agarose.

Reverse transcription was performed in a total volume of 20 μl using Transcriptor Reverse Transcriptase (Roche). Briefly, 2 μl of each RNA sample were mixed in a 0.2 ml reaction tube with 1 μl Oligo dT_18_ (Fermentas), and 10 μl of DEPC-dH_2_O, and the mixture was incubated in a PCR instrument at 65 °C for 10 min. As soon as the incubation ended, the reaction tube was cooled on ice for 2 min and then centrifuged (200 × g, 4 °C) for a few seconds. Then, 4 μl of 5 × RT buffer, 0.5 μl RNase inhibitor (Roche), 2 μl dNTP (Fermentas) and 0.5 μl Transcriptor Reverse Transcriptase were added to the reaction tube. The mixture was then incubated at 55 °C for 30 min, followed by incubation at 85 °C for 5 min before storage at −20 °C until use.

Gene-specific primers for real-time RT-PCR are listed in Table 1. Quantification of mRNA was conducted using the Mx3005P real-time PCR instrument (Stratagene, Valencia, CA). Amplification reactions were performed in a 10 μl reaction volume containing 1 μl of cDNA, 5 μl of 2 × SYBR Green Master Mix (Agilent), 0.15 μl of ROX (reference dye), 3.25 μl of RNase-free water, and 0.3 μl each of forward and reverse gene-specific primers (10 μM). Cycle amplification conditions comprised an initial denaturation step at 95 °C for 3 min followed by 40 cycles at 95 °C for 20 sec and 60 °C for 20 sec. Immediately after amplification, PCR products were analyzed by sequencing, dissociation curve analysis, and gel electrophoresis to determine specificity of the reaction. Gene expression was normalized to the glyceraldehyde 3-phosphate dehydrogenase (gapdh) internal control. All values were then expressed relative to the calibrator samples using the 2^−(ΔΔCT)^ method.

### Enzyme-linked immunosorbent assay (ELISA)

ELISA for CRH and FasL was performed using CRH Elisa kits (E03P0031) and FasL Elisa kits (E03F0051) purchased from Shanghai BlueGene Biological Technology Co., Ltd. The OD value at 450 nm was read using a microplate reader (BioTek-ELx808, BioTek Instruments, Inc.), and the results were calculated according to the standard curve.

### Immunofluorescence

All the procedures were performed at room temperature unless otherwise specified. Cumulus-free oocytes were washed 3 times in M2 medium between treatments. Oocytes were (i) fixed with 3.7% paraformaldehyde in PHEM buffer (60 mM Pipes, 25 mM Hepes, 10 mM EGTA and 4 mM MgSO4, pH 7.0) for 30 min, followed by treatment with 0.25% protease for 2 seconds to remove zona pellucida; (ii) permeabilized with 0.1% Triton X-100 in PHEM for 5 min; (iii) blocked in PHEM containing 3% BSA for 1 h; (iv) incubated at 4 °C overnight with rabbit anti-Fas (1:100, ab82419, Abcam) in 3% BSA in M2; (v) incubated for 1 h with Cy3-conjugated goat-anti-rabbit IgG (1:1000, 111-165-144, Jackson ImmunoResearch) in 3% BSA in M2; (vi) incubated for 10 min with 10 μg/ml Hoechst 33342 in M2. Negative control samples with the primary antibody omitted were also processed. The stained oocytes were mounted on glass slides and observed with a Leica laser scanning confocal microscope (TCS, SP2; Leica Microsystems). Fluorescence was detected with bandpass emission filters (Hoechst 33342, 420–480 nm; Cy3, 560–605 nm), and the captured signals for Hoechst and Cy3 were recorded as blue and red, respectively. The relative content of Fas was quantified by measuring the fluorescence intensities. For each experimental series, all high-resolution z-stack images were acquired with identical settings. The relative intensities were measured on the raw images using Image-Pro Plus software (Media Cybernetics Inc., Silver Spring, MD) under fixed thresholds across all slides. The average relative fluorescence of oocytes from the saline-injected mice was set to one, and the averages of oocytes from other treatments were expressed relative to this value.

### Data analysis

At least three replicates were used for each treatment. Percentage data were arcsine transformed and analyzed with one-way ANOVA when each measure contained more than two groups or with independent-sample t test when each measure had only two groups. A Duncan multiple comparison test was used to locate differences during ANOVA. The software used was Statistics Package for Social Sciences (SPSS 20, SPSS, Inc.). Data were expressed as means ± SEM, and P < 0.05 was considered significant.

## Additional Information

**How to cite this article**: Yuan, H.-J. *et al*. Glucocorticoids impair oocyte developmental potential by triggering apoptosis of ovarian cells via activating the Fas system. *Sci. Rep.* **6**, 24036; doi: 10.1038/srep24036 (2016).

## Figures and Tables

**Table 1:** Oligonucleotide primer sequences used for real-time PCR in this study.

cDNA	Oligonucleotide sequences (5′—3′)	Amplified product size (bp)
*Bcl2*	F: TTCGGGATGGAGTAAACTGG	157
	R: TGGATCCAAGGCTCTAGGTG	
*Bax*	F: TGCAGAGGATGATTGCTGAC	183
	R: GATCAGCTCGGGCACTTTAG	
*Igf1*	F: GGACCAGAGACCCTTTGCGGGG	210
	R: GGCTGCTTTTGTAGGCTTCAGTGG	
*Bdnf*	F: GCCTCCTCTACTCTTTCTG	255
	R: GGATTACACTTGGTCTCGT	
*GR*	F: AGTCAAGGTTTCTGCGT	233
	R: CCATCACTTTTGTTTCG	
*Gapdh*	F: AAGGTGGTGAAGCAGGCAT	244
	R: GGTCCAGGGTTTCTTACTCCT	

F, forward; R, reverse.
